# Mindfulness as a complementary intervention in the treatment of overweight and obesity in primary health care: study protocol for a randomised controlled trial

**DOI:** 10.1186/s13063-018-2639-y

**Published:** 2018-05-11

**Authors:** Vera Salvo, Jean Kristeller, Jesus Montero Marin, Adriana Sanudo, Bárbara Hatzlhoffer Lourenço, Mariana Cabral Schveitzer, Vania D’Almeida, Héctor Morillo, Suely Godoy Agostinho Gimeno, Javier Garcia-Campayo, Marcelo Demarzo

**Affiliations:** 1 DDepartment of Preventive Medicine, Federal University of São Paulo, São Paulo, Brazil; 2 Department of Psychology, Indiana State University, Terra Haute, IN USA; 3 Faculty of Health Sciences and Sports, University of Zaragoza, Zaragoza, Spain; 4 Department of Psychobiology, Universidade Federal de São Paulo, São Paulo, Brazil; 5 Primary Care, Aragon Health Sciences Institute, Zaragoza, Spain; 6 Department of Psychiatry, Miguel Servet University Hospital, University of Zaragoza, Zaragoza, Spain; 7 Hospital Israelita Albert Einstein, São Paulo, Brazil

**Keywords:** Mindfulness-Based Eating Awareness Training (MB-EAT), Obesity, Randomised controlled trial, Primary health care, Nutritional status

## Abstract

**Background:**

Mindfulness has been applied in the United States and Europe to improve physical and psychological health; however, little is known about its feasibility and efficacy in a Brazilian population. Mindfulness may also be relevant in tackling obesity and eating disorders by decreasing binge eating episodes—partly responsible for weight regain for a large number of people—and increasing awareness of emotional and other triggers for overeating. The aim of the present study protocol is to evaluate and compare the feasibility and efficacy of two mindfulness-based interventions (MBIs) addressing overweight and obesity in primary care patients: a general programme called Mindfulness-Based Health Promotion and a targeted mindful eating protocol called Mindfulness-Based Eating Awareness Training.

**Methods/design:**

A randomised controlled trial will be conducted to compare treatment as usual separately in primary care with both programmes (health promotion and mindful eating) added to treatment as usual. Two hundred forty adult women with overweight and obesity will be enrolled. The primary outcome will be an assessment of improvement in eating behaviour. Secondary outcomes will be (1) biochemical control; (2) anthropometric parameters, body composition, dietary intake and basal metabolism; and (3) levels of mindfulness, stress, depression, self-compassion and anxiety. At the end of each intervention, a focus group will be held to assess the programme’s impact on the participants’ lives, diet and health. A feasibility study on access to benefits from and importance of MBIs at primary care facilities will be conducted among primary care health care professionals and participants. Monthly maintenance sessions lasting at least 1 hour will be offered, according to each protocol, during the 3-month follow-up periods.

**Discussion:**

This clinical trial will result in more effective mindfulness-based interventions as a complementary treatment in primary care for people with overweight and obesity. If the findings of this study confirm the effectiveness of mindfulness programmes in this population, it will be possible to improve quality of life and health while optimising public resources and reaching a greater number of people. In addition, on the basis of the evaluation of the feasibility of implementing this intervention in primary care facilities, we expect to be able to suggest the intervention for incorporation into public policy.

**Trial registration:**

ClinicalTrials.gov, NCT02893150. Registered retrospectively on 30 March 2017.

**Electronic supplementary material:**

The online version of this article (10.1186/s13063-018-2639-y) contains supplementary material, which is available to authorized users.

## Background

Obesity is a problem that affects people in both developed and developing countries. Brazil has been undergoing a rapid demographic, nutritional and epidemiological transition accompanied by an increase in obesity in different population groups. In the current epidemiological setting, which has seen a decline in rates of infectious diseases, there has been a noteworthy rise in noncommunicable chronic diseases (NCDs), including obesity. With this has come increased caloric intake associated with adipose tissue gain and concomitantly a risk factor for other NCDs, such as hypertension, diabetes, cardiovascular disease and cancer. This is a critical public health problem, particularly in Brazil, where 72% of deaths are related to such diseases [1]. More than half of the Brazilian population is overweight, and obesity currently affects 20% of adults [2]. A study of food consumption between 1970 and 2009 showed a trend of increasing consumption of ultra-processed foods (baked goods, sausages, soft drinks, ready meals), and the researchers in that study also noted a reduction in the consumption of basic foods such as eggs, animal fats, fish, roots, tubers and rice, aside from the consumption of fruits and vegetables, which remained stable, albeit representing less than half of the recommended daily intake [3].

Primary health care (PHC) is fundamental for promoting, protecting and maintaining health; supporting epidemiological surveillance; and contributing to health education actions [4]. According to the World Health Organisation [5], a fundamental goal is the ability to receive and treat overweight individuals and the ability to direct care to other health units and staff in order to ensure total care of the individual with highly relevant diseases with multifactorial aetiology.

The prevention and treatment of overweight (body mass index [BMI] ≥ 25 kg/m^2^ and < 30 kg/m^2^) and obesity (BMI ≥ 30 kg/m^2^) is facilitated by an interdisciplinary approach in order to enhance the effectiveness of primary care (PC) teams. This is consistent with an increasing expansion of knowledge about the complexity of these diseases and allows for better provision of care.

A range of theoretical psychobiological approaches have addressed the underlying causes of excess food intake, which result in overweight and obesity. For example, from a psychoanalytical viewpoint, there is a linkage between a person’s needs and body limitations and the desire to obtain pleasure and avoid displeasure [6]. From a cognitive behavioural perspective, both behavioural and cognitive habits in relation to food intake develop over many years, leading to habits and food choices that become increasingly automatic [7]. In relation to the dysfunctional thoughts commonly present in overweight and obese individuals, mindfulness also has been an important tool for achieving more functional thoughts and providing better psychological health [8, 9]. When one accepts certain natural conditions of life (openness, curiosity, gentleness and non-judgement, as experienced in the mindfulness approach), it becomes easier to respond to such conditions in a more creative and functional way [9, 10]. Moreover, people with higher levels of trait mindfulness appear to have more appropriate nutritional states [11]. Mindfulness practice may also decrease inflammatory response; therefore, given that obesity is also considered an inflammatory disease, further investigation of these effects may support the value of mindfulness-based programmes [10–14].

Mindfulness may also have a particular relevance in obesity and eating disorders by reducing the episodes of binge eating, partly responsible for weight regain in a large number of people, and improving eating behaviour, as demonstrated in the Mindfulness-Based Eating Awareness Training (MB-EAT) programme by promoting awareness of unbalanced emotional states and physiological indicators generated by the process [15, 16]. Results of the meta-analyses of random effects demonstrate the medium- and long-term effects of these interventions on binge eating [17]. Multiple studies support the value of mindfulness-based interventions (MBIs) targeted at eating behaviours. They have also been adapted for use on a broader range of overweight and obese individuals with no binge eating disorders and have been shown to lead to improvement in eating behaviour and weight loss [18, 19].

### Aims

The aim of the present study is to evaluate the feasibility and relative efficacy of two MBIs by enrolling overweight and obese PC patients: the Mindfulness-Based Health Promotion (MBHP) programme and MB-EAT. The control condition will be treatment as usual (TAU).

### Hypotheses

The following are our hypotheses:

1. An MBI, regardless of whether it is a general programme or specifically designed for eating problems, improves eating behaviour if added to TAU.
2. A targeted MBI (MB-EAT) is more effective than a general MBI programme for improving eating behaviour.
3. Both MBIs (MBHP and MB-EAT) will improve mindfulness, nutritional status, biochemical parameters, self-compassion, anxiety, depression, stress and eating behaviour.
4. The provision of MBIs is feasible in the context of PC.
5. MB-EAT promotes changes other than merely treating eating behaviour.
6. The MBHP programme promotes changes in eating behaviour.
7. In both protocols, participants engage in short practices which tend to be performed even less frequently after the end of the programme.

## Methods/design

**Fig. 1:**
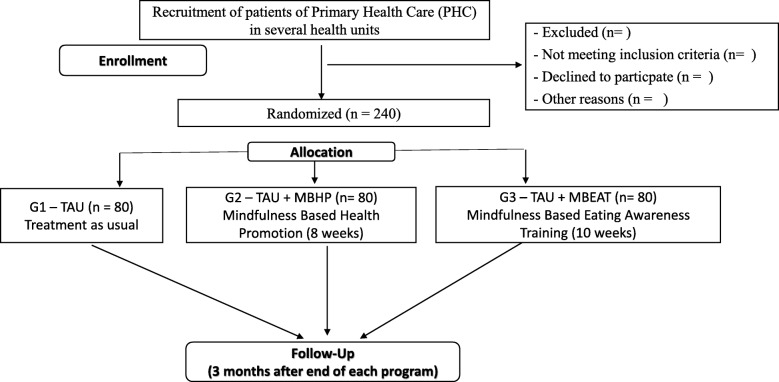
Diagram of planned study flow. *TAU* Treatment as usual, *MBHP* Mindfulness-Based Health Promotion, *MB-EAT* Mindfulness-based eating awareness training

**Fig. 2:**
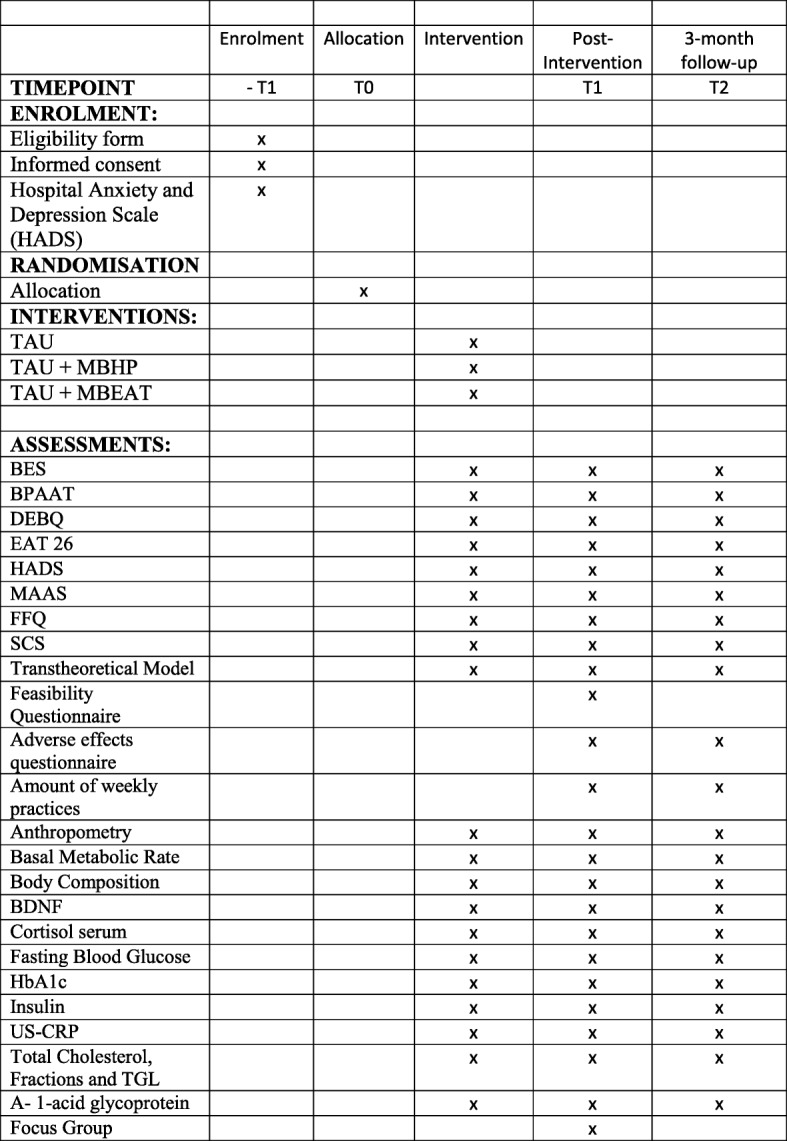
Standard Protocol Items: Recommendations for Interventional Trials (SPIRIT) diagram. Abbreviations: TAU, Treatment as usual; MBHP, Mindfulness based Health Promotion; MB-EAT, Mindfulness Based Eating Awareness Training; HADS, Hospital Anxiety and Depression Scale; BES, Binge Eating Scale; BES, Binge Eating Scale; BPAAT, Brief Physical Activity Assessment Tool; DEBQ, Dutch Food Behavior Questionnaire; EAT 26, Eating Attitude Test; FFQ, Food Frequency Questionnaire; HADS, Hospital Anxiety and Depression Scale, HBA1C, glycated hemoglobin; BDNF, Brain Derived Neurotrophic Factor; MAAS, Mindful Attention Awareness Scale; SCS, Self Compassion Scale; T1: 8 or 10 weeks Post Intervention, (TAU, TAU+ MBHP, TAU + MB-EAT); T2: 3 Months Post Intervention (TAU, TAU+ MBHP, TAU + MB-EAT); US-CRP, Ultra-sensitive C Reactive Protein

We are conducting a randomised controlled trial with stratified random allocation (nutritional status and level of overweight and obesity) of adult women and a blinded intervention status. The steps of this study will be based on the Consolidated Standards of Reporting Trials (CONSORT) [20] guidelines for reporting randomised controlled trials in a clear, transparent and comprehensive manner. These guidelines are currently endorsed by 585 international journals and more than 50% of medical journals indexed in PubMed (CONSORT). The CONSORT 2010 guideline will be used, together with an extension of CONSORT designed for applied studies [21]. The planned flow diagram of this trial is presented in Fig. 1. The protocol is reported according to the Standard Protocol Items: Recommendations for Interventional Trials (SPIRIT; Fig. 2 and Additional file 1). This study was retrospectively registered with ClinicalTrials.gov (NCT02893150) on 30 March 2017.

### Participants and setting

The sample will comprise 240 female adults with excess weight (overweight or obese) who attend PC spontaneously and of their own accord, especially in the Santo Amaro (São Paulo), and who meet the inclusion criteria for the study. Power analysis was calculated a priori using G*Power 3.1 [22] for repeated-measures analysis of variance (ANOVA). The results indicated that a sample size of 240 (*n* = 80 per group) is needed, with a significance level of 5% and a power of 80% to detect significant main effects of group and time, with a small to moderate effect size (Cohen’s *f* = 0.25), and allowing for a dropout rate of about 20%.

### Recruitment

Brazil’s publicly funded health system Sistema Único de Saúde (SUS), was created in 1988 [23]. The expansion and qualification of PHC is seen as a fundamental policy in structuring and strengthening the SUS and has been supported by the country’s Ministry of Health over the last 20 years. Several financial and organisational strategies have been used to address this challenge, notably the Family Health Strategy (FHS). The FHS consolidates a model of community-oriented PC facilities, known as basic health Units (BHUs), run by a team of nurses, general practitioners and community health workers. It includes comprehensive care, working with cultural competence and social accountability, and focuses on local families and community-based health care [24]. The FHS prioritises coverage of vulnerable low-income populations and now covers about 65% of Brazilian citizens (more than 125 million people in August 2014) [24].

The city of São Paulo, where this study will be conducted, is responsible for coordinating health care provision within its area, which is divided into six regions: Central, Western, Eastern, Northern, South-eastern, and Southern. The Southern region, in which this work will be concentrated, comprises the administrative divisions—or *subprefectures*—of Campo Limpo, Capela do Socorro, Cidade Ademar, M’Boi Mirim, Parelheiros and Santo Amaro. The Southern region, with 2.7 million inhabitants, is the most populated region in the city. One of the inclusion criteria will comprise residence in one of the following subprefectures: Campo Limpo (26 BHUs), Cidade Ademar (26 BHUs), M’Boi Mirim (31 BHUs) and Santo Amaro (5 BHUs).

An introductory lecture will be scheduled for anyone interested in participating in the research in order to point out its aims and the procedures involved. Anyone who agrees to participate in it will be required to sign an informed consent form. After the introductory lecture, evaluation tools featuring the participants’ baseline (T0) data will be applied to those who give their informed consent. The volunteers will then be randomised into one of three groups (G1, G2 or G3) and will be invited to participate in one of two programmes based on mindfulness for quality of life, lasting 10 weeks and 8 weeks (until randomisation), respectively. This is a single-blind study. The person responsible for performing the data analysis will be blind to the type of intervention. Both interventions (MBHP and MB-EAT) will be conducted by the lead researcher of this study in order to prevent inter-facilitator variability. After participants have given informed consent, they will be assessed on the basis of the inclusion and exclusion criteria. After the clarification of research procedures with the volunteers, they will then be included in a preliminary list and assigned a numeric code, which will subsequently be sent for randomisation. The participants will be randomly assigned to one of the three conditions. Randomisation will be performed by a statistician who is a member of the research team, by means of Sealed Envelope Ltd. 2017 statistics software (Sealed Envelope Ltd., London, UK) (seed 257983895669536) to allocate 15 participants to each group. The staff at the corresponding BHU will refer the participants to the research team and will also inform them of the day and time when the group sessions will be held. Allocation of participants to the groups will be performed using the restricted method to ensure balanced group sizes. The permuted block randomisation method is to be used with a 1:1:1 allocation ratio.

Participants will remain blinded to their group allocation until the first meeting. The lead researcher will be responsible for delivering both protocols and will therefore be an unblinded study member and will not be involved in the outcome analysis or data analysis (*see* Fig. 1).

As an attempt to improve adherence to the programme, all groups will receive an invitation to a monthly maintenance session (according to each protocol after the end of the programme, at 8 or 10 weeks) in addition to weekly emails with suggestions regarding formal and informal practices. Adherence to mindfulness practices by groups G2 and G3 will be evaluated at T1 and T2 with an appropriate form. Attendance will be monitored at each session; any individual who has not participated in at least half + 1 of the programme’s total sessions will be considered a loss to dropout. Each week the participants will be invited to keep a practice journal, and average practice at home, type of practice, frequency and so forth will be verified in their own form in the post-intervention evaluation and follow-up.

### Inclusion criteria

The following are the inclusion criteria:

- Women aged ≥ 18 and < 60 years
- Literate
- BMI ≥ 25 kg/m^2^ and < 40 kg/m^2^
- An interest in the aims of this study and provision of informed consented to be randomised to one of the three groups (G1 = control or TAU, G2 = MBHP + TAU, or G3 = MB-EAT + TAU)

### Exclusion criteria

The following are the exclusion criteria:

- Pharmacological treatment for obesity
- BMI ≥ 40 kg/m^2^, because individuals in this state are usually already receiving specialised services (ambulatory or hospital setting) and medication
- Cushing syndrome
- Pregnant or intending to become pregnant within the following 6 months
- Pacemaker bearers
- Illiterate
- A substance use issue (drugs and/or alcohol)
- Untreated hypothyroidism or hyperthyroidism
- Acute-phase depression (< 6 months with depression), diagnosed with schizophrenia or psychotic disorders, use of drugs that cause cognitive attentional and concentration losses (such as powerful anti-anxiety drugs)
- Practitioners of mindfulness, meditation, yoga or similar practices in the previous 6 months (with formal practice at least once per week);
- Any type of bariatric surgery

### Measures

#### Socio-demographic health and quality of life

Data to be collected include age, sex, marital status, education level, professional status, number of hours of sleep, morbidity profile and bowel habits.

##### Eating Attitude Test

The Eating Attitude Test indicates the presence of abnormal eating patterns and provides an index of the severity of typical concerns of patients with eating disorders. The instrument consists of 26 items, with 6 response options (always, very often, often, sometimes, rarely and never), with factor analysis performed on these items in three aspects (diet, bulimia nervosa and oral control) [25].

##### Dutch Food Behaviour Questionnaire

The Dutch Food Behaviour Questionnaire (DBEQ) is a self-applied questionnaire validated by Wardle [26] and adapted and translated into Portuguese by Almeida [27]. The questionnaire comprises 33 items assessed on a scale of 5 points (never=1, rarely=2, sometimes=3, often=4, very often=5), spread across three scales: the scale of restriction includes 10 items; the external intake range also consists of 10 items; and the scale of emotional intake consists of 13 items.

- *Restricted food intake*: the participants’ eating style and knowledge of nutritional habits and the effort they regularly exert to control their appetite and food intake.
- *Emotional eating*: related to the person’s emotional state, it represents the loss of control over food intake due to exposure to factors of emotional stress, which results in food disinhibition.
- *Food intake due to external factors*: eating influenced by how attractive the flavour and taste of food is; it will represent the disinhibition or loss of control that occurs due to external factors intrinsic to the food available or the social situation in which they are offered.

##### Binge Eating Scale

The Binge Eating Scale (BES) is a self-applied questionnaire developed by Gormally et al. [28] that is widely used in English-speaking countries and has been translated into Portuguese for use in Brazil by Freitas et al. [29]. It shows how to appropriately discriminate obese individuals according to the severity of their binge eating. It consists of a list of 16 items assessed on a Likert scale. Every statement matches a number from 0 to 3 points, ranging from the absence (“0”) to the maximum severity (“3”) of binge eating. The final score is the result of the sum of the points for each item. Scoring is as follows: ≤ 17 = normal; 17–30 = variation in tendency to eat a lot (anything really); ≥ 30 or more = binge eating.

##### Mindfulness skills

The Mindful Attention Awareness Scale evaluates the level of mindfulness and presents a one-dimensional structure with 15 items, answered using a Likert scale of 6 points [10]. It is the most widely used mindfulness scale in research on the subject and is validated for use in Brazil [30].

##### Self-Compassion Scale

The Self-Compassion Scale contains 26 items that measure the feeling of self-compassion through positive factors such as self-kindness and gentleness, state of mindfulness and a feeling of belonging to humanity, as well as negative factors such as self-judgement, isolation and excessive self- identification [31]. This scale is validated in Portuguese [32].

Compassion allows the person to have a non-judgemental attitude and acceptance of her or his own mistakes, as well as an understanding, affection and kindness towards oneself and one’s failures [33–35]. Individuals with obesity often have feelings of guilt and a lack of care and kindness towards themselves, which impairs the success of any attempts at dietary treatment and weight control.

##### Hospital Anxiety and Depression Scale

The Hospital Anxiety and Depression Scale (HADS) of Zigmond and Snaith [36] consists of 14 items. Seven of these are for evaluation of anxiety (HADS-A), and the remaining seven form a depression scale (HADS-D). The scale of measurement is 4 points, ranging from 0 to 3, with each scale reaching 21 points. Marcolino et al. [37] recommended a score ≥ 9 as a cut-off point for both subscales. The following definitions are attributed to scores on both scales:

- HAD-A/D: 0–8, without anxiety; ≥ 9, with anxiety
- HAD-A/D: 9–10, mild
- HAD-A/D: 11–14, moderate
- HAD-A/D: 15–21, severe

##### Brief Physical Activity Assessment Tool

The Brief Physical Activity Assessment Tool is a questionnaire administered by a health care professional. It comprises two questions that measure the frequency and duration of physical activity and its intensity, both vigorous and moderate, during a typical week. The scoring system identifies people who are active enough (performing at least three sessions per week of 20 minutes each with vigorous intensity, at least five sessions per week of 30 minutes with moderate intensity or at least five sessions of any combination of physical activities, moderate or vigorous) [38, 39]. Physical activity is also to be evaluated as a categorical variable, according to type of activity, frequency and duration.

##### Transtheoretical Model (Stages of Change)

The Transtheoretical Model (Stages of Change) theory views behaviour change as a process in five stages based on the behaviour demonstrated by the person and the person’s intention to change. These stages are as follows:

1. Precontemplation: The person is not ready to consider change.
2. Contemplation: There is awareness of the need for behaviour change but no real commitment in this direction.
3. Preparation: There is an intention to change and act in about 1 month.
4. Action: Specific changes occur with intent to change lifestyle behaviours.
5. Maintenance: The changes have been achieved, and the focus is on avoiding relapses [40].

An intervention based on the Transtheoretical Model will help to promote anthropometric and nutritional improvements [41]. In this work, we will use the proposition by the Brazilian Health Ministry [42, 43] for the evaluation of the stages of change regarding weight loss in order to check the participants’ adherence to the treatment in the different intervention groups and the control group.

##### Dietary intake

Dietary intake will be evaluated by applying the Food Frequency Questionnaire for obesity, which has previously been validated [44]. This questionnaire will be used to evaluate dietary intake from a qualitative point of view, according to the different food groups and how frequently they are consumed before, prior to and after the intervention and at follow-up.

##### Anthropometric assessment

The anthropometric assessment will be composed of collecting weight, height and waist circumference measurements. The participants will be classified according to BMI using the World Health Organisation cut-off points [45].

##### Body composition

Body composition will be assessed by bioelectrical impedance analysis using a BIA 450 version 5.1 device (Biodynamics, Shoreline, WA, USA), which emits a low-intensity electrical current (800 μA to 50 kHz) that runs through the person’s body to measure resistance, reactance and phase angle [46]. The volunteers will be instructed on pre-test procedures following the manufacturer’s recommendations. Bioelectrical impedance analysis will require a total evaluation time of approximately 1 minute.

##### Basal metabolic rate

Basal metabolic rate will be evaluated by use of MetaCheck calorimeter version 2.05 (KORR Medical Technologies, Salt Lake City, UT, USA). Indirect calorimetry is a safe, practical and non-invasive method which uses a portable device. This technique measures energy expenditure by analysing the volume of oxygen consumed (VO_2_), the volume of carbon dioxide produced (VCO_2_) and the respiratory quotient (VO_2_/VCO_2_), which enables the amount of energy needed for metabolic processes to be assessed. This evaluation will require a total time of approximately 15 minutes [47, 48].

#### Biochemical analysis

All tests (fasting blood glucose, haemoglobin A1c, insulin, total cholesterol, fractions and triglycerides, serum cortisol and ultra-sensitive C-reactive protein will be performed and samples collected between 7 and 9 a.m. according to the procedures described by the São Paulo Municipal Health Department [49] at each of the BHUs participating in the research. Brain-derived neurotrophic factor (BDNF) will be measured using the enzyme-linked immunosorbent assay method. BDNF has been associated with various aspects of plasticity in the brain. Stress-induced remodelling of the cortex, hippocampus and amygdala coincides with changes in BDNF levels. It is known that some forms of mindfulness meditation training can reduce stress-induced cortisol secretion, which could have neuroprotective effects by increasing levels of BDNF [50]. Samples for alpha-1-glycoprotein analysis (marker of inflammation) will be collected at baseline and at 8 or 10 weeks (according to the type of intervention) as well as 3 months post-intervention at the outpatient clinic of the Universidade Federal de São Paulo on the same day as completion of psychometric scales, food consumption and body composition evaluations. In addition to the quantitative analysis, this study will include qualitative evaluation; data will be gathered from focal groups, with open-ended questions about the expectations of the programme, the viability and implications in an everyday context, and changes in eating behaviour after the end of the intervention.

### Follow-up assessments

The evaluations for the main outcome (eating behaviour) and the secondary outcomes (mindfulness skills, self-compassion, nutritional status, depression and stress) will occur at baseline (T0), immediately after the 8- or 10-week intervention (T1) and at 3 months post-intervention (T2) (*see* Table 1). The outcomes of the practices, unexpected (adverse) effects and maintenance of and adherence to practices will all be evaluated in the T1–T2 intervals. The estimated time for completing the questionnaires and other evaluations is approximately 90 minutes, and the questionnaires will be administered before the beginning of the first and last sessions of each group, as well as at follow-up. For groups G2 and G3, one session will be offered each month to cultivate and strengthen the practice of mindfulness in the group, according to the practices described in each protocol. After the 3-month follow-up, the control group will be offered what is determined to be the most effective intervention, either MB-EAT or MBHP (Table 1).

**Table 1:** Overview of measures and corresponding measurement time points

Measure	Target concept	Baseline	T1	T2
BES	Binge eating	x	x	x
BPAAT	Physical activity	x	x	x
DEBQ	Food style	x	x	x
EAT-26	Presence of abnormal eating patterns	x	x	x
HADS	Anxiety and depression symptoms	x	x	x
MAAS	Mindfulness skills	x	x	x
FFQ	Food Frequency Questionnaire	x	x	x
SCS	Self-compassion	x	x	x
Transtheoretical Model	Behaviour change	x	x	x
Feasibility Questionnaire	Feasibility of intervention		x	
Adverse effects questionnaire	Occurrence adverse effects		x	x
Amount of weekly practices	Adherence to practice		x	x
Anthropometry	Change in weight	x	x	x
Basal metabolic rate	Basal metabolism	x	x	x
Body composition	Fat mass and lean mass	x	x	x
BDNF	Brain-derived neurotrophic factor	x	x	x
Cortisol serum	Stress	x	x	x
Fasting blood glucose	Glycaemic control	x	x	x
HbA1c	Haemoglobin A1c	x	x	x
Insulin	Insulin	x	x	x
US-CRP	Ultra-sensitive C-reactive protein	x	x	x
Total cholesterol, fractions and TGL	Lipid profile	x	x	x
Alpha-1-glycoprotein	Inflammatory process	x	x	x
Focus group	Qualitative analysis		x	x

*Abbreviations: BES* Binge Eating Scale, *BPAAT* Brief Physical Activity Assessment Tool, *DEBQ* Dutch Food Behaviour Questionnaire, *EAT-26* Eating Attitude Test, *FFQ* Food Frequency Questionnaire, *HADS* Hospital Anxiety and Depression Scale *HbA1c* glycated haemoglobin, *MAAS* Mindful Attention Awareness Scale, *SCS* Self-Compassion Scale, *T1* 8 or 10 weeks post-intervention (TAU, TAU + MBHP, TAU + MB-EAT), *T2* 3 months post-intervention (TAU, TAU + MBHP, TAU + MB-EAT), *US-CRP* Ultra-sensitive C-reactive protein, *BDNF* Brain-derived neurotrophic factor, *TGL* Triglycerides

### Interventions

#### TAU

According to nutritional status, overweight or obesity, as well as the presence of co-morbidity, different actions can comprise the treatment offered in PC [1]. For individuals presenting with overweight (BMI 25–29.9 kg/m^2^) but with no co-morbidities, PC teams at the different BHUs will organise care plans to enable them to achieve a normal BMI range (BMI 18.5–24.9 kg/m^2^).

Aside from the participants’ inclusion in group activities, for those who have co-morbidities such as hypertension and diabetes, individual dietary prescriptions will be offered by a nutritionist if deemed necessary. This decision will be discussed between the PC and matrix support teams.

For treatment of obesity (BMI 30–40 kg/m^2^) with or without co-morbidities, PC teams from the corresponding BHUs and matrix support teams must assess the need for the provision of behavioural therapy and pharmacotherapy, and must organise it, when appropriate, because these are usually provided within the PC system. Group activities can be offered to promote healthy eating and physical exercise. The more complex cases or cases with a BMI ≥ 40 kg/m^2^ will be assisted by specialised services (ambulatory or hospital setting).

The MBHP and MB-EAT groups will be led by a researcher who has experience in both protocols and specific meditation training and practice. The MBHP group will have a minimum of 8 participants and a maximum of 20; the MB-EAT group will have between 8 and 15 participants.

#### Mindfulness-Based Health Promotion + TAU

Our research groups (in Brazil and Spain) developed a general MBI that is an 8-week programme called Mindfulness-Based Health Promotion (MBHP) [51]. It is based on the original model developed by Kabat-Zinn et al. (mindfulness-based stress reduction [MBSR]) [52, 53] and subsequently adapted by our research group to better fit the context and needs of PHC as well as the local and national health systems [54]. It has been applied by the “Mente Aberta” Centre in Brazil (www.mindfulnessbrasil.com) and by the University of Zaragoza in Spain (www.webmindfulness.com). The sixth of these sessions is conducted in silence for the purpose of deepening the practice of mindfulness [51] and is similar to the MBSR retreat day session, but it is adapted to make it more feasible to implement in health centres and facilities. This more didactic and clearer sequence of the content helps improve the training of PHC providers, such as mindfulness teachers, enabling them to offer the programme to patients independently (cascading effect) [51].

As with MBSR, participants receive suggestions for activities to be performed at home or in the workplace on a regular daily basis, aiming for 15 to 20 minutes of practice, on average, and up to 45 minutes if possible (for those who are more engaged and motivated). They are also encouraged to bring the experience of mindfulness into their daily lives (informal practices). The main mindfulness practices used are mindfulness of breathing, mindfulness of the body (body scan), mindful walking and mindful movements (in which gentle physical movements are used), which can be practised by people with different levels of physical capacity. In addition to the MBSR protocol, the MBHP introduces a few dynamic activities to gain better insight into the concepts developed in the psychoeducational part of the programme, such as the “first and second suffering” and “hi-thanks-goodbye” practices; the practice of kind awareness (addressing equanimity and interpersonal aspects, based on Buddhist practices of compassion and self-compassion); and the 3-minute breathing space practice of mindfulness (from the mindfulness-based cognitive therapy protocol) [55]. The two last practices are adapted from the Breathworks Institute protocol [56].

#### MB-EAT + TAU

Inspired by the MBSR programme and eating-related research, MB-EAT was initially developed for individuals with compulsive eating problems and obesity [57]. The programme includes the four main techniques of meditation mentioned in the MBHP programme. In addition, mindfulness is applied to various aspects of the participants’ eating behaviour and to their experience of inner wisdom and outer wisdom. Every session also includes mindfulness practices for the cultivation of a greater awareness of hunger, fullness, taste awareness with different foods, and triggers for overeating. Overall, a greater sense of self-awareness and self-acceptance regarding eating and weight is cultivated. The specific elements of each session in both programmes are described in Table 2.

**Table 2:** Content and formal practices of each session of MBHP and MB-EAT

Program	MBHP	MB-EAT
session		
1	What is mindfulness? *Formal practices:* -Eating a raisin -Body scan	Internal and external wisdom *Formal practices:* - General mindfulness meditation - Eating four raisins - Introduction to a general mindfulness practice
2	Mindfulness in breathing *Formal practices:* -Mindfulness while breathing -Body scan	Mini-meditations, eat consciously and food calories (smart changes) *Formal practices:* - General mindfulness meditation - Introduction to mini-meditations (10 min) - How to read labels and the nutritional composition of products - Mindfully eating high-fat food: cheese and crackers
3	Mindfulness in the body (part I) *Formal practices:* -Mindful walking -Mindfulness while breathing	Mindfulness while breathing Awareness of physical hunger, awareness of body and emotional eating *Formal practices:* - Longer meditation practice - Hunger awareness self-rating practice - Body scan
4	Mindfulness in the body (part II) *Formal practices:* - Mindful movements - Mindfulness while breathing, sensations, sounds and thoughts - 3-Min breathing space in pairs - Mindful walking	Physical activity, satisfaction of taste and self-healing touch *Formal practices:* - General mindfulness meditation - Taste satiety exercise with chocolate food - Healing self-touch
5	Mindfulness and acceptance *Formal practices:* - Mindful movements - Mindfulness of thoughts - 3-Min breathing space in doubles	Awareness of fullness, conscious choices and forgiveness *Formal practices:* - General mindfulness meditation - Stomach fullness and body satiety - Making mindful choices: cookies and chips - Emotional eating, anger and forgiveness meditation
6	Silence *Formal practices:* - Mindful movements - Body scan - Mindfulness while breathing - Mindful walking	Integrating internal and external wisdom: becoming conscious about nutrition and energy balance *Formal practices:* - Mindful eating choices exercise: fruits and veggies - Integrated mindful eating meditation
7	Compassion *Formal practices:* - Metta (for oneself and others) - Mindfulness while breathing - Mindful movements	Eating, conscious choices and community meal *Formal practices:* - Brief meditation practice - Mindful choice/taste awareness meditation - Mindful eating: potluck meal
8	Mindfulness for life *Formal practices:* - Metta (for oneself and others) - Mindfulness poem	Conscious movement and emotions *Formal practices:* - Mindfulness meditation - Body scan - Trigger meditation - Craving meditation - Walking meditation
9		Stress, suffering, eating and values: working with thoughts and emotions *Formal practices:* - Mindfulness meditation - My favourite food exercise
10		Breaking the chain, connecting to yourself and moving on *Formal practices:* - Meditation practice - Experience of sitting meditation - Wisdom meditation - Shared celebration/eating

Both the MBHP and MB-EAT programmes also assign practices to be used at home during the week, including mindfulness-based meditation and eating-related practices for MB-EAT. Emails will be sent to participants after each session, encouraging them to engage in the practice of the week, as well as to those who fail to provide reasons for their absence from the programme. The monthly maintenance sessions offered may stimulate continued practice at home by participants in groups G2 and G3.

### Analysis plan

#### Primary outcome measures

The primary outcome variables are those indicating improvement in eating behaviour. Self-report instruments to be used to track eating behaviour are the DEBQ and the BES, collected from T0 to T2.

### Secondary outcome measures

Secondary outcome measures include greater self-knowledge, the perception of emotional triggers for overeating, increase in self-compassion resulting in higher self-care and better adherence to treatment, improvement of nutritional status (reduction of body weight by ≥ 5%) and biochemical examinations throughout the intervention, as well as maintenance of this status (without weight regain) during a 3-month follow-up period. This maintenance could lead to the prevention of multiple morbidities related to excess body weight. Levels of mindfulness, stress, anxiety (psychometric scale and serum cortisol) and self-compassion will be assessed. Body composition, basal metabolism and food intake, as well as maintenance of practices and the feasibility of the programme, will be also evaluated. Changes in lifestyles and eating patterns of participants in each group will be evaluated at the end of the programme and 3 months after the intervention.

### Data handling

All study-related information will be stored in secure folders with limited access. Paper-based data collection forms will contain only participant numbers to maintain confidentiality and will be stored in a locked cabinet in an area with limited access. Electronic data files will be password-protected (RedCap). The list linking participant numbers and personal information will be stored in a separate password-protected file. Paper-based data entry will be double-checked. Data will be stored for 5 years after the end of inclusion. The study has low negligibility risks; therefore, no data monitoring committee will be assigned. Only the first author and lead researchers, or persons assigned by them, will have access to the final dataset. Authors of the final trial report will make substantial contributions to the design, conduct, interpretation and reporting of the trial. Study results will be presented via publications and presentations; study participants, funders and involved clinicians will receive a summary of the study results.

### Data analysis

The qualitative variables will be described as numbers and percentages, and quantitative data will be summarised as mean values with SD if the assumption of normality of data has been satisfied; otherwise, the median and 25% and 75% percentiles will be used. The three conditions for the treatment will be compared with baseline measures to evaluate the success of randomisation. In this case, the chi-square test or Fisher’s exact test for qualitative variables will be used, and one-way ANOVA or the Kruskal-Wallis test will be applied for quantitative variables.

Longitudinal data (T0, T1 and T2) will be collected for each group, which means that they will not be independent; therefore, repeated-measures ANOVA will be used. When assessing the effectiveness of the TAU programme, repeated-measures ANOVA will be used to assess the main effects of time and treatment, and the interaction between them. The analysis will be performed with the complete sample, and we plan to perform analyses in subgroups stratified by BMI. The models will be adjusted for possible confounding variables, such as age, BMI and other relevant covariates. Differences will be identified by using test linear hypotheses after ANOVA, and multiple testing will be adjusted by the Bonferroni correction.

The data will be evaluated for the intention-to-treat analysis; that is, the analysis will include all participants after randomisation. For the per-protocol analysis, women in the intervention groups who do not participate in at least four sessions will be excluded. For the attrition analysis, we will compare women who complete the protocol with women who answered only the baseline questionnaire.

All data analysis will be performed with STATA version 14.2 software (StataCorp, College Station, TX, USA) [58]. *p* Values < 0.05 will be considered statistically significant. No datasets were generated or analysed during the present study.

### Qualitative assessment

At the end of each protocol/intervention, focus groups consisting of a minimum of 4 and a maximum of 15 volunteer participants will be conducted and incorporated into the programmed sessions (G2 and G3). The focus groups will be fully recorded in digital audio, with participant observation of a team member. The focus group will be held through structured interviews to investigate whether the programme made a difference in the participants’ health and nutritional status. Qualitative data will be analysed on the basis of focus group. Verbatim reporting will identify emerging themes from the qualitative content analysis [59]. We expect this analysis to identify the reasons for adherence/non-adherence to the mindfulness and TAU programmes and the health effects of the programmes.

## Discussion

Most people do not want to go on a diet, because they usually have an emotional relationship with food. This gives mindful eating an advantage because it is a non-diet approach, which is less unnerving for patients, and more realistic, doable and durable by enhancing self-acceptance and acceptance of the body, without an attachment to the number of calories in each food. Several studies and meta-analysis reviews [17, 60–64] indicate that mindful eating can be an important tool in weight loss and maintenance, without the need to count calories, which is usually recommended by the Academy of Nutrition and Dietetics.

MB-EAT was adapted from MBSR and specifically developed for compulsive eating disorders and related problems [57]. The main objective of the MB-EAT is to control the balance between physiological and non-nutritional factors that influence eating behaviour, helping individuals to cultivate a greater awareness of hunger and satiety as well as awareness of arising emotional states and external triggers (*see* Table 2).

The programme emphasises the pleasure and affection related to food while encouraging healthy standards when choosing food, in relation to both the quantity and the quality of food ingested. MB-EAT is an effective way to internalise and maintain behaviour changes. This programme emphasises the importance of mindfulness meditation in cultivating the ability to focus one’s attention [65].

People are more convinced by the information they discover on their own than by what is brought to them by others; it is already known that manipulating the cognitive field alone is not enough to change eating behaviours or habits. More serious than the maintenance of obesity is the “yo-yo pattern” promoted by a low adherence to dietary treatments, resulting in constant weight regain, which aggravates metabolic problems and poses a great challenge to health professionals. This therefore requires new intervention strategies and research.

Weight maintenance is also associated with factors such as the manifestation of intrinsic motivation, such as greater self-confidence regarding weight loss, better strategies of reaction and the ability to cope with stress (decentring, metacognition), feelings of self-efficacy, autonomy, healthy narcissism and greater stability and psychological strength [66, 67]. Mindfulness is able to act positively in this direction, and both the MBIs, which present reduction in weight gain, hunger and emotional eating [61], as well as MB-EAT, which seems to be effective in the United States and Europe for stress [68, 69], anxiety reduction [70, 71] and body weight issues [72–75], must have their findings replicated.

Research into mindful eating is more recent than research into the mindfulness stress reduction programmes. There is a very limited number of clinical trials, which are predominantly based on psychometric scales, often with few samples. Reviews concerning mindful eating have used different protocols, with a waiting list as a control, and an active control used only a few times. Follow-up is even rarer, and in most cases it does not reach 6 months.

Few studies have provided significant data on at-home mindfulness practice (quantity and quality) in MBIs, particularly in mindful eating programmes. Studies of this nature are generally scarce, and they are non-existent in Brazil. Further research with more objective measures, aside from the psychometric scales, should be carried out to clarify the mechanisms involved in these benefits [62, 63] and to identify the most effective protocol for this population.

The present study, which uses psychometric, anthropometric and biochemical parameters, as well as dietary intake, will not be limited to a quantitative approach and could address the gaps left by psychometric scales. This information is as yet unpublished in Brazil and, as far as we know, is not published in any international literature either. This mixed-methods (quantitative and qualitative) study could point to more objective forms of effective intervention, contributing to cost reduction and therefore to more sustainable weight loss, with less weight regain, and consequently to a reduction in excess weight-related co-morbidities that burden the health care system.

The feasibility assessment for the implementation of the mindfulness programme provided for in this study is one of the steps for the medium- and long-term implementation of this intervention in PC. PC is the main gateway for patients in a health system, and with the inclusion of meditation in the national policy of integral and complementary practices in 2017 by the Brazilian Ministry of Health, it is believed that more resources will be dedicated to the sector, thus allowing professionals to qualify as teachers. In addition, different types of professional training have been offered for a period of 2 years in the most varied protocols, which would allow the inclusion of more people interested in working with mindfulness. The adherence of the participants to the programme, the acceptance of the professionals and cost-effectiveness measures are future steps that should be contemplated for implementation to become reality [18].

This study presents certain limitations, which are as follows. São Paulo is a large metropolis, with an extensive surface area. Covering one BHU per week, even restricted to the Southern region, will require a great deal of travel mileage. Added to this will be the characteristic transit problems inherent in a large city, which requires 1.5–2 hours to arrive at each BHU. Further studies should be carried out covering BHUs in other regions, with both sexes and including the elderly population, in order to ensure greater representativeness and generalisability. This is a very deprived population living in peripheral areas with scarce financial resources and low schooling, which adds more difficulty to the understanding and completion of questionnaires, possibly leading to errors. Although in this study we will evaluate the same health region, with the same guidelines applied, there is heterogeneity of treatment between BHUs.

### Trial status

At the time of initial manuscript submission, recruitment was already in progress (starting March 2018) but was not yet complete (prospectively expecting completion in May 2019). This study protocol reports protocol version 1 (dated 26 Nov 2017).

## Additional file

Additional file 1: SPIRIT checklist. (DOC 137 kb)

## Abbreviations

ANOVA: Analysis of variance
BDNF: Brain-derived neurotrophic factor
BES: Binge Eating Scale
BHU: Basic health unit
BMI: Body mass index
BPAAT: Brief Physical Activity Assessment Tool
CONSORT: Consolidated Standards of Reporting Trials
DEBQ: Dutch Eating Behaviour Questionnaire
EAT-26: Eating Attitude Test
FFQ: Food Frequency Questionnaire
FHS: Family Health Strategy
HADS: Hospital Anxiety and Depression Scale
HbA1C: Haemoglobin A1c
MAAS: Mindful Attention Awareness Scale
MB-EAT: Mindfulness-based eating awareness training
MBHP: Mindfulness-Based Health Promotion
MBI: Mindfulness-based intervention
MBSR: Mindfulness-based stress reduction
NCD: Noncommunicable chronic disease
PC: Primary care
PHC: Primary health care
SCS: Self-Compassion Scale
SPIRIT: Standard Protocol Items: Recommendations for Interventional Trials
SUS: Sistema Único de Saúde
TAU: Treatment as usual
US-CRP: Ultra-sensitive C-reactive protein
VCO_2_: Volume of carbon dioxide produced
VO_2_: Volume of oxygen consumed
